# The very early evolution of protein translocation across membranes

**DOI:** 10.1371/journal.pcbi.1008623

**Published:** 2021-03-08

**Authors:** AJ Harris, Aaron David Goldman

**Affiliations:** 1 Key Laboratory of Plant Resources Conservation and Sustainable Utilization, South China Botanical Garden, Chinese Academy of Sciences, Guangzhou, China; 2 Department of Biology, Oberlin College and Conservatory, K123 Science Center, Oberlin, Ohio, United States of America; 3 Blue Marble Space Institute of Science, Seattle, Washington, United States of America; Weizmann Institute of Science, ISRAEL

## Abstract

In this study, we used a computational approach to investigate the early evolutionary history of a system of proteins that, together, embed and translocate other proteins across cell membranes. Cell membranes comprise the basis for cellularity, which is an ancient, fundamental organizing principle shared by all organisms and a key innovation in the evolution of life on Earth. Two related requirements for cellularity are that organisms are able to both embed proteins into membranes and translocate proteins across membranes. One system that accomplishes these tasks is the signal recognition particle (SRP) system, in which the core protein components are the paralogs, FtsY and Ffh. Complementary to the SRP system is the Sec translocation channel, in which the primary channel-forming protein is SecY. We performed phylogenetic analyses that strongly supported prior inferences that FtsY, Ffh, and SecY were all present by the time of the last universal common ancestor of life, the LUCA, and that the ancestor of FtsY and Ffh existed before the LUCA. Further, we combined ancestral sequence reconstruction and protein structure and function prediction to show that the LUCA had an SRP system and Sec translocation channel that were similar to those of extant organisms. We also show that the ancestor of Ffh and FtsY that predated the LUCA was more similar to FtsY than Ffh but could still have comprised a rudimentary protein translocation system on its own. Duplication of the ancestor of FtsY and Ffh facilitated the specialization of FtsY as a membrane bound receptor and Ffh as a cytoplasmic protein that could bind nascent proteins with specific membrane-targeting signal sequences. Finally, we analyzed amino acid frequencies in our ancestral sequence reconstructions to infer that the ancestral Ffh/FtsY protein likely arose prior to or just after the completion of the canonical genetic code. Taken together, our results offer a window into the very early evolutionary history of cellularity.

## Author summary

Cellularity is an ancient, fundamental organizing principle of life. Central to cellularity is the cell membrane, which separates a cell from the outside environment. Cell membranes contain proteins that perform a range of functions including transport of compounds across the membrane barrier, sensing the external environment, and performing certain metabolic activities that must occur in proximity to the membrane. Therefore, embedding proteins into membranes and secreting proteins across membranes is an essential aspect of cellularity, not to mention an essential aspect of life itself. One cellular system that accomplishes embedding proteins into membranes and secreting proteins across membranes is the signal recognition particle (SRP) system. The SRP system has a core consisting of the proteins, FtsY and Ffh, which derive from a single FtsY/Ffh ancestral protein. The system is also associated with a protein-based passageway, the Sec channel, for embedding proteins within the membrane or allowing them to pass through it. To study the SRP system and the central protein of the Sec channel, SecY, in early life, we reconstructed evolutionary trees from protein sequences. Based on these trees, we infer that the last universal common ancestor (LUCA) of life had an SRP system and SecY channel that were similar to those in extant organisms, while an earlier ancestor of the LUCA possessed a more rudimentary system for embedding and secreting proteins. Moreover, the ancestral Ffh/FtsY protein probably arose prior to or soon after the final amino acids were added to the standard genetic code.

## Introduction

The emergence of cellular organisms from non-cellular replicators is considered one of the major transitions, or key innovations, in evolutionary history [1] that may have been the pre-requisite for the earliest speciation events [2] and for subsequent colonization of all the habitable environments on Earth [3,4]. Cellular organisms must build and maintain a cell membrane as well as both populate it with integral membrane proteins and secrete proteins out of it. In very early cellular life forms, the ability to embed proteins within the cell membrane would have been essential for a number of important biological processes, such as controlled cell division and any metabolism that required the import of large metabolites and that was reliant on ATP synthesis by proton motive force.

**Fig 1:**
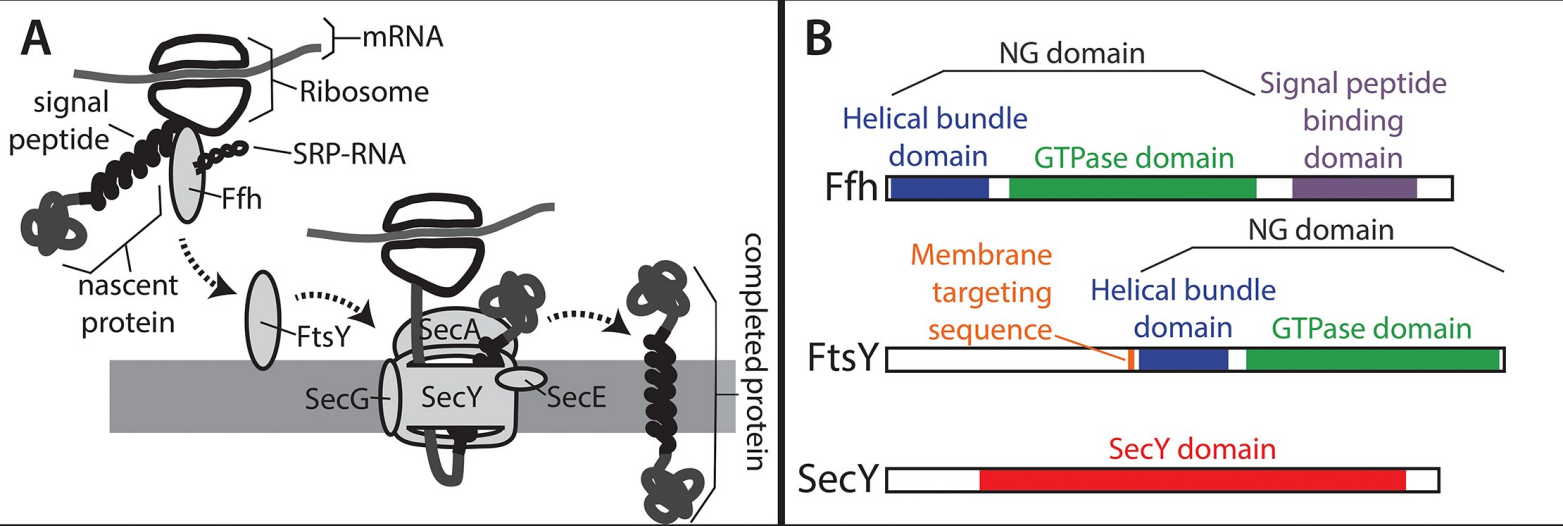
An overview of the canonical SRP/Sec membrane translocation system. SRP is responsible for binding signal peptides in nascent proteins and delivering the nascent protein and ribosome to the membrane surface. A) A signal peptide within the nascent protein is bound by Ffh. Ffh forms a complex on the membrane surface with FtsY and SRP-RNA along with the nascent protein and the ribosome that is synthesizing it. After the SRP complex is formed, the signal peptide on the nascent protein enters the Sec channel and newly added protein is translocated across the membrane while it is being synthesized. Upon release of the protein into the membrane, the signal peptide becomes a transmembrane domain. The Sec channel is a multiprotein complex composed of three proteins, SecYEG. Many auxiliary proteins promote this process, most notably SecA, which associates with SecYEG and drives translocation through the channel via ATP hydrolysis. B) Domain architectures of the three proteins analyzed in this study are shown based on their E. coli homologs. Both Ffh (Uniprot ID = P0AGD7) and FtsY (Uniprot ID = P10121) contain a a helical bundle domain (blue) and a GTPase domain (green), which together are called the NG domain. Ffh also contains a region called the M domain (purple), which binds the signal sequence of a nascent protein. FtsY also contains a membrane targeting sequence, or MTS, domain which binds to the surface of the membrane (orange). SecY (Uniprot ID P0AGA2) contains up to ten predicted transmembrane domains, but only one domain annotated by the pfam database, called “SecY” (red).

While several different systems have evolved that facilitate protein translocation into and across membranes, the signal recognition particle (SRP) system and its associated Sec secretion channel (illustrated in Fig 1) are particularly ubiquitous across the tree of life. The SRP system relies on two distinct, paralogous proteins. One of these proteins is a cytosolic protein known as Ffh in Bacteria and SRP54 in Archaea and Eukarya (hereafter Ffh) that binds a signal sequence in a nascent protein and guides the ribosome synthesizing the protein to the membrane [5–7]. The other protein is a membrane bound receptor for the Ffh-ribosome complex that is known as FtsY in Bacteria and SRα or SR receptor in Archaea and Eukarya (hereafter FtsY) [8–10]. In many Bacteria, the SRP system comprises only these two proteins and an RNA called SRP RNA, which facilitates the assembly and disassembly of the protein-synthesizing complex at the membrane [11,12]. In Archaea and Eukarya, the system includes these three components as well as other accessory proteins that are bound to Ffh and FtsY [13].

Ffh and FtsY belong to the SIMIBI class of proteins, which consist of dimerizing pairs that form GTPase domains, or G domains, at their interfaces [14–16]. Ffh and FtsY also each possess a helical bundle domain, which is situated in close proximity to the GTPase domain and is sometimes referred to as the N domain because it is closer to the N-terminus of the protein than the GTPase domain. Together, the helical bundle and GTPase domains comprise a conserved region shared by both FtsY and Ffh and are often collectively referred to as the NG domain. Ffh also possesses a C-terminal, methionine-rich M domain [17,18], while FtsY has a membrane targeting sequence (MTS) domain within or upstream of the helical bundle domain [19,20].

A protein that is targeted to the membrane by the SRP system will contain at least one signal peptide sequence. Once the ribosome synthesizes the signal sequence, Ffh will bind to it through the M domain [17,18], and this typically pauses the translation process. Subsequently, the ribosome-bound Ffh forms a heterodimer with FtsY on the membrane surface. The interaction between Ffh and FtsY is likely assisted by the SRP RNA in response to signal peptide binding in the M domain of Ffh [21]. In Bacteria and Archaea, the complex comprising FtsY, Ffh, SRP RNA, and the ribosome and nascent protein, forms on the interior surface of the plasma membrane (or the inner plasma membrane in Gram-negative bacteria), while in eukaryotes, it occurs on the exterior surface of the endoplasmic reticulum. Though FtsY is located on the surface of the membrane, it is not bound to the membrane through a typical hydrophobic transmembrane domain. Instead, the MTS domain is composed of positively charged amino acids, which bind to negative charges in phospholipid head groups on the surface of the membrane [22].

The interaction between Ffh and FtsY facilitates the transfer of the signal peptide sequence from Ffh to a translocation channel called SecYEG in Bacteria and Archaea, and Sec61 in eukaryotes (for review, see [23]). Within the translocation system, SecY is the largest protein subunit and forms the actual transmembrane channel. It is the only Sec subunit known to be present throughout the tree of life [24]. In addition to the channel, the Sec system as it has been described in *E*. *coli* also includes a protein that drives protein translocation by way of ATP hydrolysis, SecA, and a handful of other auxiliary proteins that promote the function of the channel or are used in special cases [25]. Via the translocation system, the signal sequence is transferred to the interior of the membrane and translation resumes, with the new sequence being fed through the translocation channel. A protein that is destined to be secreted through the membrane usually contains an N-terminal signal sequence that is removed once it has been synthesized by a signal peptidase protein. Proteins that are destined to be embedded in the membrane contain one or more internal signal sequences that become transmembrane domains in the finished protein. After transfer of the signal peptide to the translocation channel, the Ffh/FtsY/SRP RNA complex dissociates. This dissociation is triggered by the hydrolysis of GTP molecules [26,27] that are bound at the interface of the Ffh-FtsY heterodimer through the GTPase domains contained in both proteins [14–16].

Both the SRP system and the SecY protein family are regarded as ancient and their nearly ubiquitous presence in extant organisms suggests that they had originated at least by the time of the last universal common ancestor of life, the LUCA [28]. However, there is no comprehensive protein phylogeny of the SRP system with systematic, robust taxonomic sampling and there is no current phylogeny of SecY (but see [29]) that takes into account recent advances in biodiversity discovery, taxonomic classifications, and newly available genome sequences of archaeal and bacterial species [30–33]. Therefore, in this study, we performed phylogenetic analyses of the SRP system and SecY proteins to elucidate the very early evolutionary history of these families. Our reconstructed phylogeny indicates that ancestors of Ffh, FtsY, and SecY were all present by the time of the LUCA. Furthermore, within the phylogeny, the Ffh and FtsY subtrees are connected by a well-supported pre-LUCA branch, supporting the notion that this protein family predates the origins of the LUCA. Based on the phylogeny, we used ancestral sequence reconstructions combined with protein function prediction to show that the ancestral Ffh and FtsY protein that predated the LUCA (hereafter, ancestral Ffh/FtsY) potentially arose before the completion of the genetic code, but that it likely contained many functional components of the modern SRP system allowing it to facilitate a rudimentary form of protein translocation and secretion across cell membranes. Together, these results provide a framework for understanding the very early evolution of membrane translocation, a foundational system required by all cellular organisms.

## Results and discussion

### Survey of SRP and Sec proteins across Bacteria and Archaea

**Fig 2:**
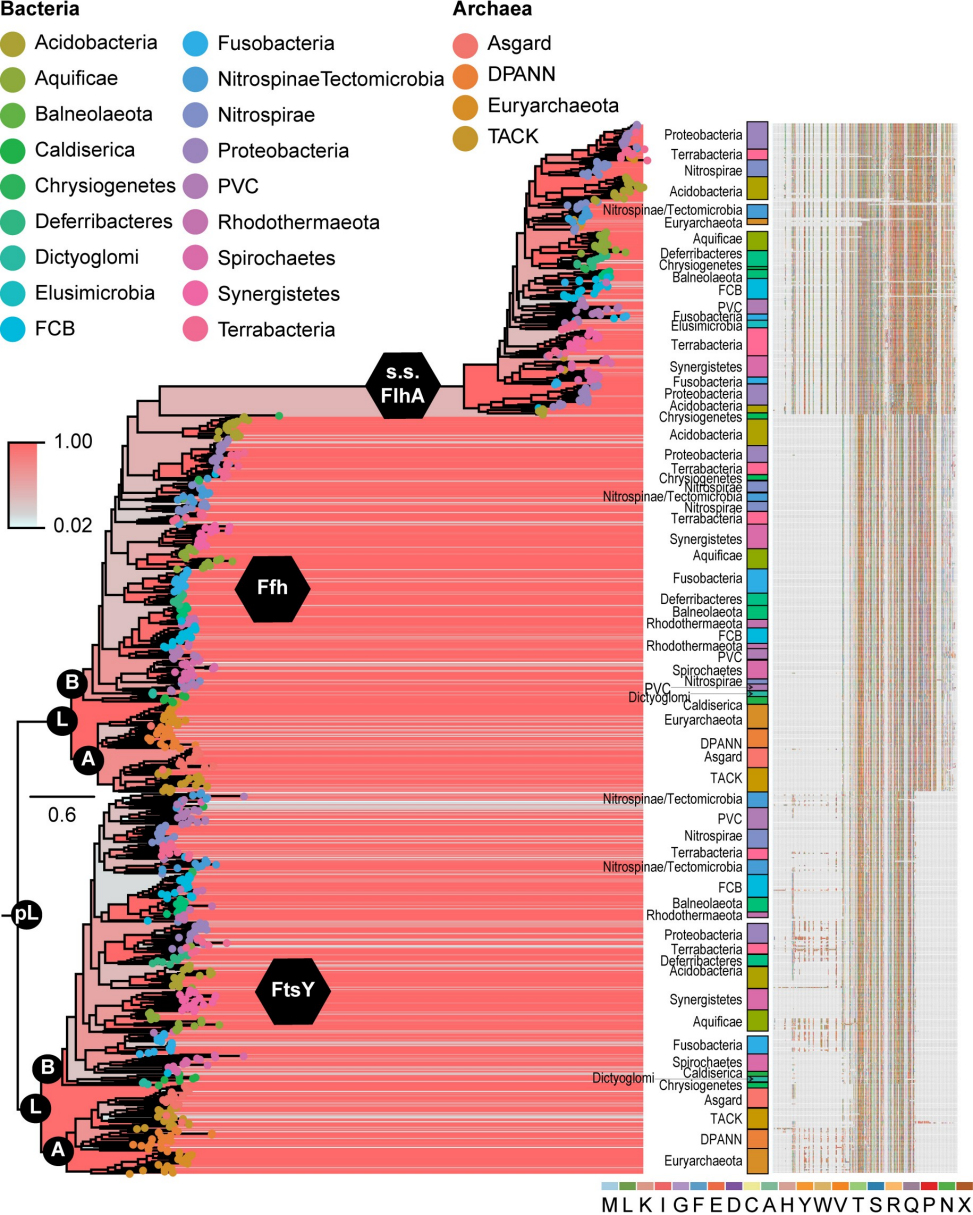
Maximum clade credibility tree resulting from Bayesian analysis of accessions of SRP system proteins, FtsY and Ffh, plus the flagellar protein, FlhA, and the type III secretion system proteins, EscV, YscV, and HrcV. Support values are indicated by color from red (higher) to blue (lower). Labeling of internal nodes indicates ancestral Ffh/FtsY (pre-LUCA; pL), the LUCA (L), and crown clades of Bacteria (B) and Archaea (A). Major clades comprising phyla or superphyla of Bacteria or Archaea are labeled to the right of terminals, notwithstanding one or a few nested accessions of other groups. The multiple sequence alignment from which we generated the phylogeny is shown to the far right. The complete phylogeny and multiple sequence alignment are available as supplementary files in newick and fasta formats, respectively.

We identified homologs of FtsY, Ffh, and SecY according to protein BLAST searches (BLASTp; [34]) and selected 20 sequences or the maximum number available (whichever was smaller; see Materials and Methods) from each bacterial and archaeal phylum or super phylum for inclusion in downstream analyses. Our final protein dataset for the SRP system comprised 938 protein sequences representing 18 bacterial and four archaeal phyla and superphyla. Of these protein sequences, 348 were of FtsY and 330 were of Ffh. Additional accessions represented two other paralogous protein families identified using BLASTp and occurring primarily within a subset of Bacterial lineages. Of these additional accessions, 46 comprised type III secretion system proteins and 214 represented the flagellar protein, FlhA (see explanation in Methods). The alignment of all 938 sequences consisted of 1666 characters (Fig 2 and S3 File) and had 21.1% average overall pairwise identity and 36.2% average positive pairwise identity based on the BLOSUM62 scoring matrix. Almost all bacterial and archaeal phyla and superphyla that were well-represented in the Genbank protein database were also well-represented in our dataset. The only exception was the bacterial phylum, Elusimicrobia, for which we obtained only three accessions; one each for FtsY, Ffh, and the type III secretion system. Other phyla and superphyla that were less well-represented in Genbank generally yielded fewer than 20 sequences.

For SecY, we obtained 355 protein sequences representing the same taxonomic diversity of Bacteria and Archaea as for the SRP system. The sequences of SecY were available at roughly the rates expected based on taxonomic representation in Genbank, and Elusimicrobia was not under-represented. The alignment of these 355 sequences comprised 835 characters (Fig 2 and S4 File) and had an overall pairwise identity of 33.1% and a pairwise identity of 51.6% based on BLOSUM62 scoring.

We also attempted to use similar methods to obtain sequences of signal peptidase proteins, which cleave N-terminal signal sequences from translocated proteins. Previous literature has indicated that Signal Peptidase I, called LepB in Bacteria, was present in the LUCA [35]. One COG from the eggNOG database [36], COG0681, contains homologs of Signal Peptidase I and is found in both Bacteria and Archaea. However, a BLASTp search of *H*. *volcanii* proteins using LepB *E*. *coli* as a query yielded a poor match (query coverage = 19%, e value = 0.28) that failed a reciprocal best hit test. An additional search of the entire Uniprot database of archaeal proteins using LepB of *E*. *coli* as a query yielded only short hits, with the top hit consisting of only 30 amino acids.

We obtained a similar result for Signal Peptidase II, called LpsA in Bacteria. Signal Peptidase II is represented by eggNOG cluster COG0597, which contains both bacterial and archaeal proteins. A BLASTp search of *H*. *volcanii* proteins using a sequence of LpsA from *E*. *coli* as a query yielded no hits at all. However, a search of the entire Uniprot database of archaeal proteins using *E*. *coli* LpsA yielded a top hit to a putative protein from an archaean, *Halobellus sp*. *Atlit-31R* (NCBI Taxonomic ID = 2282130) that passed a reciprocal best hit test against the *E*. *coli* genome. When we applied this *Halobellus* protein as a BLAST query against the entire Uniprot database of archaeal proteins, we obtained only 82 significant hits under our e-value threshold. None of these protein sequences represented reviewed proteins, and all of them were from uncultured or unclassified taxa. Even if these results represent archaeal orthologs of LpsA, their scarcity is more consistent with a few horizontal gene transfer events than inheritance of Signal Peptidase II by descent from the LUCA. Therefore, neither Signal Peptidase I nor Signal Peptidase II warranted further phylogenetic analysis.

Additionally, we sought to determine if SecA of the Sec translocation channel may have been present in the LUCA. SecA drives the translocation of proteins through the membrane via ATP hydrolysis. SecA belongs to the eggNOG cluster, COG0653, which contains both bacterial sequences and archaeal sequences. The sequence of SecA protein from *E*. *coli* did not have a detectable homolog in *H*. *volcanii* based on a BLASTp search. A search of the entire Uniprot database of archaeal proteins using SecA of *E*. *coli* yielded a top hit to a putative protein from an unclassified archaean, Natrialbaceae archaeon XQ-INN 246 (NCBI Taxonomic ID = 2419781), that passed a reciprocal best hit test against the *E*. *coli* genome. However, this protein from Natrialbaceae yielded only 56 significant BLAST hits when used as a query against the entire Uniprot database of archaeal proteins, and many of the hits were very short compared to the query. None of these protein sequences represented reviewed proteins. As with Signal Peptidase II, if these results represent orthologs, their scarcity is more consistent with a few horizontal gene transfer events. Thus, we determined that the SecA family did not merit further phylogenetic analysis in this study.

### Phylogenetic evidence for early ancestors of Ffh, FtsY, and SecY

The Bayesian phylogenetic reconstruction for the SRP system showed a generally well-resolved tree with two major lineages comprising FtsY and Ffh, each with clear bacterial and archaeal clades (Fig 2 and S5 File). This tree, therefore, provides very strong evidence that an ancestral version of Ffh and an ancestral version of FtsY were both present in the genome of the LUCA and that a common ancestor of both proteins, an ancestral Ffh/FtsY, was present prior to the LUCA. Additionally, a mixed clade of FlhA and type III secretion system proteins arose within the bacterial Ffh clade on a very long evolutionary branch. Neither the type III secretion system proteins nor FlhA proteins were resolved as monophyletic groups, which is also consistent with mixed clusters of these proteins obtained in Clustal Omega (S1 File; e.g., cluster 2).

Within the phylogeny of the SRP system proteins, most protein accessions representing individual phyla and superphyla formed monophyletic groups. Additionally, many relationships between superphyla and phyla were consistent between subtrees of FtsY and Ffh, such as the relationship between the Asgard and TACK archaeal lineages, between the bacterial lineages of Aquificae and Synergistetes, as well as among Acidobacteria, Proteobacteria, and Terrabacteria. However, relationships within the clade of FlhA and type III secretion system proteins were less consistent with the other two subtrees. Across the entire tree, there was little phylogenetic evidence for horizontal gene transfers across taxonomic domains; namely, only one archaeal lineage was nested within the clade of bacterial FlhA and type III secretion system proteins.

**Fig 3:**
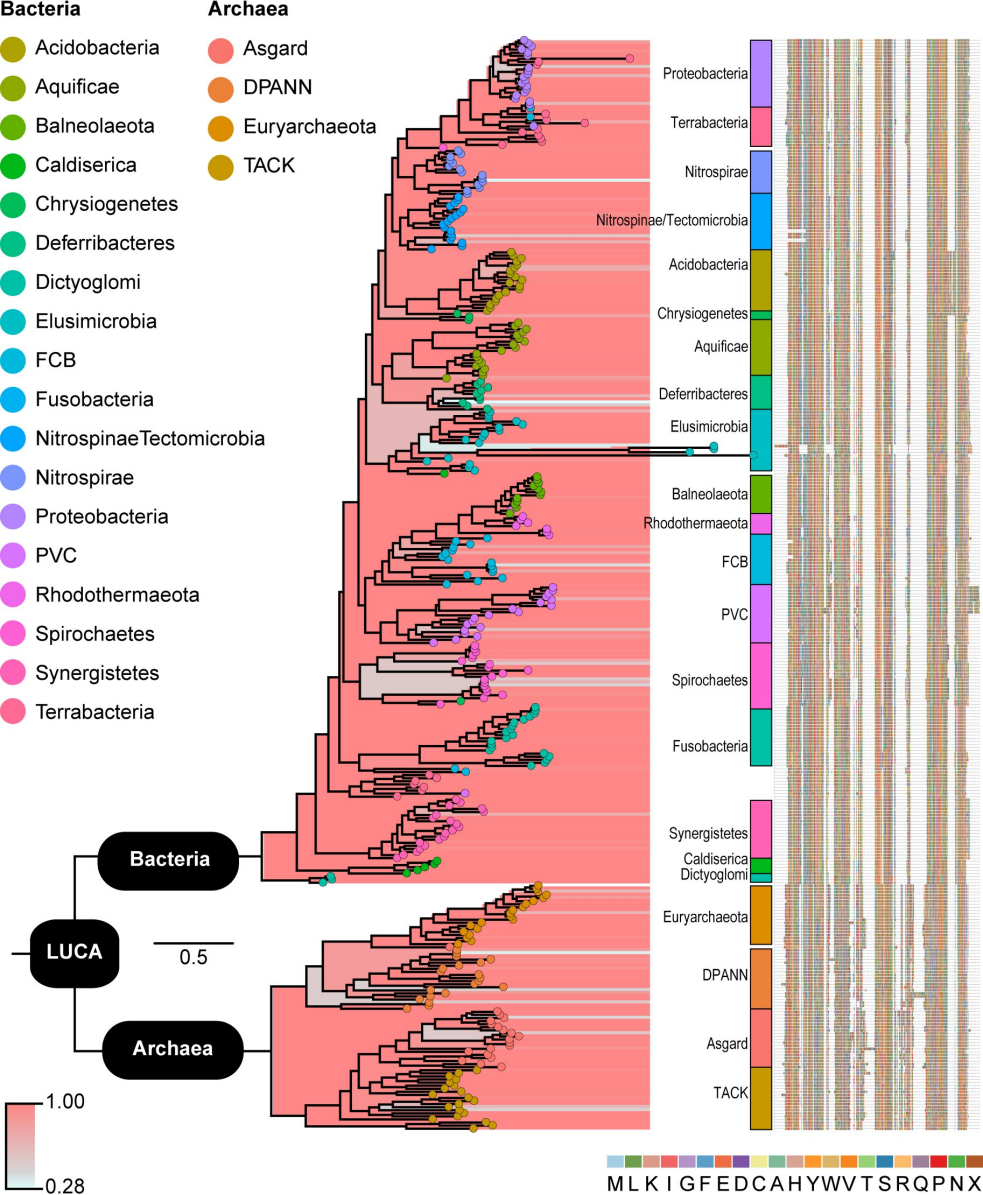
Maximum clade credibility tree resulting from Bayesian analysis of accessions of SecY. Support values are indicated by color from red (higher) to blue (lower). Major clades comprising phyla or superphyla of Bacteria or Archaea are labeled to the right of terminals, notwithstanding one or a few nested accessions of other groups. The multiple sequence alignment from which we generated the phylogeny is shown to the far right. The complete phylogeny and multiple sequence alignment are available as supplementary files in newick and fasta formats, respectively.

The phylogenetic reconstruction of SecY resulted in a very highly supported tree (Fig 3 and S6 File). The tree consisted of two major clades corresponding to the bacterial and archaeal domains, therefore representing robust evidence for an ancestor of SecY being present in the genome of the LUCA. Within the tree, most bacterial and archaeal phyla and superphyla formed clades, and the longest branches were in the Elusimicrobia clade. Relationships among phyla and superphyla were somewhat inconsistent with those in the FtsY and Ffh subtrees. For example, accessions representing Aquificae and Synergistetes were only distantly related. However, similarities are also abundant, such as Caldiserica and Dictyoglomi being among the earliest diverging lineages in both the SecY and Ffh/FtsY trees, and accessions of Terrabacteria and Proteobacteria being closely related. We observed no phylogenetic evidence in the SecY tree of horizontal gene transfers across taxonomic domains.

Beyond the principal results that SecY was present in the LUCA and an ancestor of Ffh/FtsY existed prior to the LUCA, we observed an interesting trend within the Elusimicrobia phylum. Specifically, we found that few Elusimicrobia possess homologs of the SRP system and that several Elusimicrobia accessions of SecY were on long phylogenetic branches, which suggest considerable, possibly rapid, evolutionary change (Fig 3). It is possible that loss of FtsY and Ffh in some Elusimicrobia has driven the evolution of SecY to compensate, such as to enable SecY to interact with other proteins that have taken over the tasks of the SRP system. Our results indicate that more targeted studies of the SRP system and SecY in Elusimicrobia may be merited.

### Sequence-based characterization of ancestral SRP system proteins and SecY

Our phylogenetic results clearly support the antiquity of both the Ffh/FtsY and the SecY protein families. Therefore, we sought to further examine the early evolution of these proteins by characterizing the ancestral sequences corresponding to ancient nodes within their respective phylogenetic trees. To accomplish this, we performed ancestral state reconstructions to infer the sequences of early Ffh, FtsY, and SecY proteins. Ancestral state reconstruction does not, on its own, provide a guide for the inclusion of indels, or gaps, in the reconstructed sequences. Therefore, we reconstructed ancestral sequences using a broad range of probability thresholds for inferring amino acids versus gaps.

The sequence reconstructions of the Ffh and FtsY proteins in the LUCA, as well as of the ancestral Ffh/FtsY protein, all predictably varied in length based on the threshold for including gaps (S7 and S8 Files). With a 90% probability threshold for inferring gaps, the average lengths of 100 reconstructed sequences in the SRP system were 763 (SD: 33.80) for ancestral Ffh/FtsY and, in the LUCA, 681 (SD: 118.10) for FtsY and 665 (SD: 124.14) for Ffh. With only a 10% threshold, the averages were 289 (SD: 28.98), 427 (SD: 125.57), and 435 (SD: 5.48) respectively. For the 50% threshold, the values were intermediate between these.

The sequences of ancestral Ffh/FtsY obtained using the 10%, 50%, and 90% thresholds for inferring gaps all possessed helical bundle, GTPase, and MTS domains, while the sequences reconstructed using the 50% and 90% cut-offs also included extended N- and C-termini. An extended C-terminal region is typical of Ffh proteins and includes the M domain that contains a signal peptide binding motif. We recovered this extended C-terminal region in the sequence of ancestral Ffh/FtsY protein reconstructed using a 90% threshold for indel inclusion. An extended N-terminal region occurs in some FtsY proteins, but it appears to have arisen several times independently in evolutionary history based on our phylogeny and is not conserved in either length or sequence (Fig 2, S3 File). Therefore, unsurprisingly, the N-terminal region of ancestral Ffh/FtsY obtained using the 90% threshold did not contain any homologs to domains or motifs within the PFAM database [37,38]. Overall, the 10% threshold for indel inclusion yielded a sequence for ancestral Ffh/FtsY that most closely resembled the NG domain shared among all modern Ffh and FtsY proteins, so we chose this sequence for downstream analyses except as noted.

**Fig 4:**
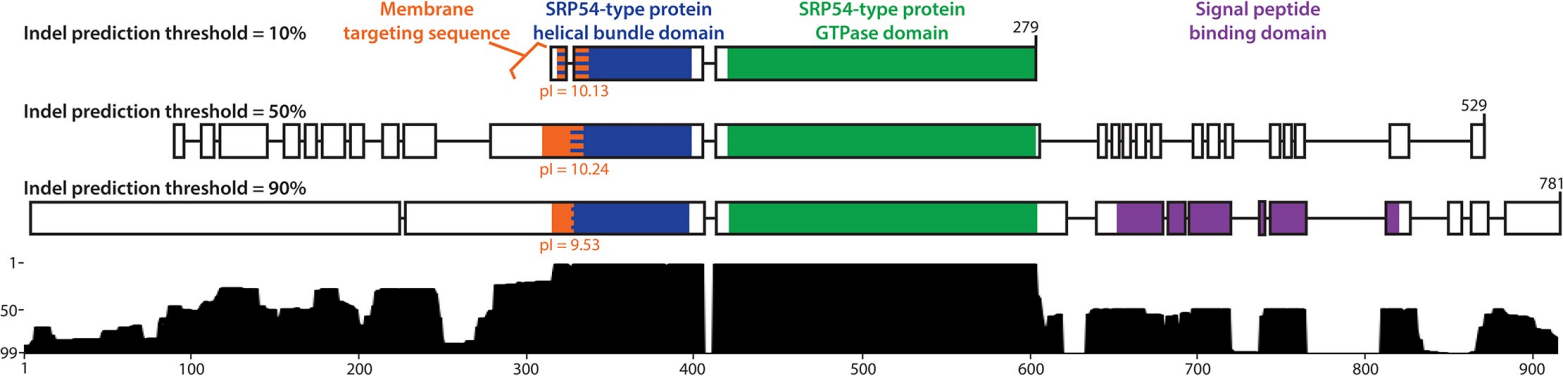
Functional annotation of ancestral Ffh/FtsY. Domains and motifs from the Pfam Database are mapped onto sequences of the reconstructions of ancestral Ffh/FtsY using three different thresholds for inference of gaps (top sequence = 10%, middle sequence = 50%, and bottom sequence = 90%). The histogram below represents the frequency that an amino acid (as opposed to a gap) appears in each position across all thresholds for inferences of gaps (1%-99%). There is agreement across all three sequence reconstructions that ancestral Ffh/FtsY contained the GTPase domain (green) and the four helical bundle domain (blue) that are typical of both Ffh and FtsY proteins. Sequence alignment to *E*. *coli* Ffh (see Methods) revealed an MTS domain (orange) in all three reconstructed ancestral sequences with characteristically basic isoelectric points. In addition to these domains, the ancestral sequence that was reconstructed using a 90% threshold for inference of gaps contains a C-terminal peptide binding domain (purple), but this domain is typical only of modern Ffh proteins and is not observed in modern FtsY proteins.

Domains and motifs of the sequence reconstruction resulting from the 10% indel threshold show that the ancestral Ffh/FtsY protein very likely contained the helical bundle (e-value: 1.4e-76) and GTPase domains (e-value: 1.7e-15; Table 1 and Fig 4) that are found in modern Ffh and FtsY proteins. The helical bundle domain of ancestral Ffh/FtsY also appears to contain a membrane targeting sequence (MTS) motif in its N-terminal helix that has an isoelectric point similar to a recently characterized MTS motif in *E*. *coli* (10.13 and 12.01, respectively). Thus, this suggests that the ancestral Ffh/FtsY protein may have been able to bind to membranes through electrostatic attraction as in modern FtsY proteins. Similar to the ancestral Ffh/FtsY and modern proteins, the Ffh and FtsY of the LUCA both possessed a helical bundle domain (e-values: 8.0e-21, 7.2e-11, respectively) and a GTPase domain (e-values: 1.3e-77, 1.6e-76, respectively) (Table 1). The sequence reconstruction for Ffh was also inferred to possess a C-terminal M domain (e-value: 8.7e-43; Table 1).

**Table 1:** Sequence-based function prediction for ancestors of Ffh, FtsY, and SecY.

Protein	Inferred Origin	PFAM (alignment locus|e-value)	Protein localization based on SOSUI (hydrophobicity index)
**Ancestral Ffh/FtsY**	pre-LUCA	SRP54-type protein, helical bundle domain (6–81|1.4e-76) SRP54-type protein, GTPase domain (95–279|1.7e-15)	cytoplasmic (0.0792)
**Ffh**	LUCA	SRP54-type protein, helical bundle domain (5–80|8.0e-21) SRP54-type protein, GTPase domain (94–278|1.3e-77) Signal peptide binding domain (309–408|8.7e-43)	cytoplasmic (-0.3597)
**FtsY**	LUCA	SRP54-type protein, helical bundle domain (96–175|7.2e-11) SRP54-type protein, GTPase domain (189–373| 1.6e-76)	cytoplasmic (-0.4542)
**SecY**	LUCA	SecY translocase (58–377|4.9e-101)	trans-membrane(0.9908)

In addition to characterizing the functional domains and motifs within these ancestral proteins, we also used SOSUI [39] to investigate whether they were most likely to be cytoplasmic (i.e., soluble in an aqueous solvent) or embedded within a membrane. SOSUI predicts whether a protein is located in the membrane or is cytoplasmic based on its chemical properties and constituent amino acids, especially focusing on hydrophobicity [39]. Analyses in SOSUI showed that the reconstructed sequence of ancestral Ffh/FtsY as well as FtsY and Ffh of the LUCA were cytoplasmic proteins with average hydrophilicity indices of 0.0792, 0.4541, and -0.3597, respectively (Table 1). Note that while FtsY is membrane bound, it is not an integral membrane protein, and is therefore expected to have a hydrophilicity index typical of cytoplasmic proteins.

For the ancestor of SecY within the LUCA, the average lengths of reconstructed sequences were 393 (SD 0.00), 598 (SD: 24.44), and 696 (SD: 23.45) for the 10%, 50%, and 90% thresholds for inferences of gaps. We used the 10% threshold for downstream analyses to avoid biases in our treatments of this protein family compared to the FtsY and Ffh ancestors and because the unaligned sequence lengths that were recovered using the 10% threshold showed no variation among 100 reconstructions, suggesting strong support for the placement of gaps among the results. The sole PFAM annotation for the reconstructed SecY in the LUCA shows that the majority of this ancestral sequence aligns to the SecY translocase domain family (e-value: 4.9e-101; Table 1). Moreover, the reconstructed sequence of SecY for the LUCA was predicted to be a membrane protein with an average hydrophobicity of 0.9908 (Table 1) based on SOSUI, and this is consistent with SecY in modern species.

### Structure-based characterization of ancestral SRP system proteins

In order to further characterize the functions of the ancestral Ffh/FtsY protein as well as Ffh and FtsY of the LUCA, we performed protein structure prediction and structure-based function prediction on the reconstructed sequences using I-TASSER [40], a highly accurate method that performs both of these tasks. The predicted structure for the ancestral Ffh/FtsY protein in I-TASSER showed similarities to known structures of FtsY and Ffh from all three domains of extant life that have been solved by X-ray diffraction (e.g., PDB IDs 3ndb, 3dm5, 2og2, and 4ak9; [18,41,42]). Proteins from the SRP systems of extant organisms with the greatest predicted structural similarity to the inferred ancestral protein were Ffh proteins from the archaean, *Methanocaldococcus jannaschii* (Jones et al. 1984) Whitman 2002 strain DSM 2661 (RMSD = 1.16; PDB ID 3ndbB) and *Pyrococcus furiosus* Erauso *et al*. 1993 (RMSD = 1.37; PDB ID 3dm5A), and the bacterium, *Thermus aquaticus* Brock & Freeze, 1969 (RMSD = 1.94; PDB ID 1ng1). Structure-based functional characterization (Table 2) predicted that ancestral Ffh/FtsY could bind GTP (GO:0005525), SRP RNA (GO:0008312), and proteins (GO:0005515) and would be capable of nucleoside-triphosphatase activity (GO:0017111).

**Table 2:** Structure and structure-based function prediction for ancestors of Ffh, FtsY, and SecY.

Protein	Inferred Origin	Most Structurally Similar Protein (PDB accession)	RMSD score	Molecular Function (GO term|score)	Biological Process (GO term|score)	Cellular Component (GO term|score)
**FtsY-Ffh Ancestor**	pre-LUCA	Signal recognition 54 kDa protein (3NDBB)	1.16	GTP binding (GO:0005525| 1.00) nucleoside-triphosphatase activity (GO:0017111|1.00) 7S RNA binding (GO:0008312|0.98) protein binding (GO:0005515|0.92)	SRP-dependent cotranslational protein targeting to membran (GO:0006614|1.00) cell division (GO:0051301|0.71) cell cycle (GO:0007049|0.71)	signal recognition particle, endoplasmic reticulum targeting (GO:0005786|0.98) plasma membrane (GO:0005886|0.71)
**Ffh**	LUCA	Signal recognition 54 kDa protein (3DM5A)	0.67	GTP binding (GO:0005525|0.91) nucleoside-triphosphatase activity (GO:0017111|0.86) 7S RNA binding (GO:0008312|0.69) protein binding (GO:0005515|0.32)	SRP-dependent cotranslational protein targeting to membrane (GO:0006614|0.91)	signal recognition particle, endoplasmic reticulum targetin (GO:0005786|0.69)
**FtsY**	LUCA	SRP receptor alpha subunit (6FRKy)	2.24	7S RNA binding (GO:0008312|0.92) GTPase activity (GO:0003924|0.61) signaling receptor activity (GO:0038023|0.41) protein binding (GO:0005515|0.40) GTP binding (GO:0005525|0.37)	SRP-dependent cotranslational protein targeting to membrane(GO:0006614|0.92) cell division (GO:0051301|0.63) cell cycle (GO:0007049|0.63)	intrinsic component of plasma membrane (GO:0031226|0.40) cytosol (GO:0005829|0.40) signal recognition particle, endoplasmic reticulum targeting (GO:0005786|0.37)
**SecY**	LUCA	Protein translocase subunit SecY (5NCOg)	1.07	P-P-bond-hydrolysis-driven protein transmembrane transporter activity (GO:0015450|0.95) protein binding (GO:0005515|0.43) signal sequence binding (GO:0005048|0.43)	transmembrane transport (GO:0055085|0.92) SRP-dependent cotranslational protein targeting to membrane (GO:0006614|0.43)	integral component of membrane (GO:0016021|0.92) plasma membrane (GO:0005886|0.73) Ssh1 translocon complex (GO:0071261|0.43)

1 Top scoring GO terms from among consensus terms

Ancestral Ffh/FtsY was also predicted to be localized to the plasma membrane (GO:0005886) just as FtsY of modern species is (S1 Table). However, in contrast to both FtsY and Ffh of modern species (e.g., *E*. *coli* and *H*. *volcanii*; S1 Table), I-TASSER did not predict any GO terms suggesting cytosolic or cytoplasmic localization of ancestral Ffh/FtsY. Additionally, the predicted structure of ancestral Ffh/FtsY showed unexpected predictions for the biological processes of cell division (GO:0051301) and the cell cycle (GO:0007049), which are not annotated for FtsY and Ffh sequences of representative modern species (S1 Table). Within the LUCA, structural predictions suggest that both FtsY and Ffh facilitated cotranslational protein targeting to the cell membrane (GO:0006614), and FtsY is predicted to be localized to both a cytosolic and membrane component of the cell (GO:0005829, GO:0031226, respectively). No cellular component predictions for Ffh were recovered among the consensus GO terms produced by I-TASSER. Similar to ancestral Ffh/FtsY, the FtsY ancestor in the LUCA is also predicted to have functions in cell division and the cell cycle.

The predictions that the pre-LUCA Ffh/FtsY ancestor and FtsY in the LUCA are associated with cell division and the cell cycle are probably spurious. They come from local structural similarities to well-characterized proteins. While there is a direct relationship between protein structure and molecular function, there is only an indirect relationship between protein structure and the biological processes that the protein is associated with. That said, while FtsY of modern species is generally not known to operate directly within the cell cycle (S1 Table; [43]), it occurs in an operon with two vital cell division proteins, FtsE and FtsX, in some bacterial species, such as *E*. *coli*. Notably, an evolutionary explanation for the inclusion of FtsY within the *ftsYEX* operon is lacking [43,44]. Thus, it is possible that FtsY had an ancient function in cell division in addition to its role in membrane translocation and embedding of proteins.

**Fig 5:**
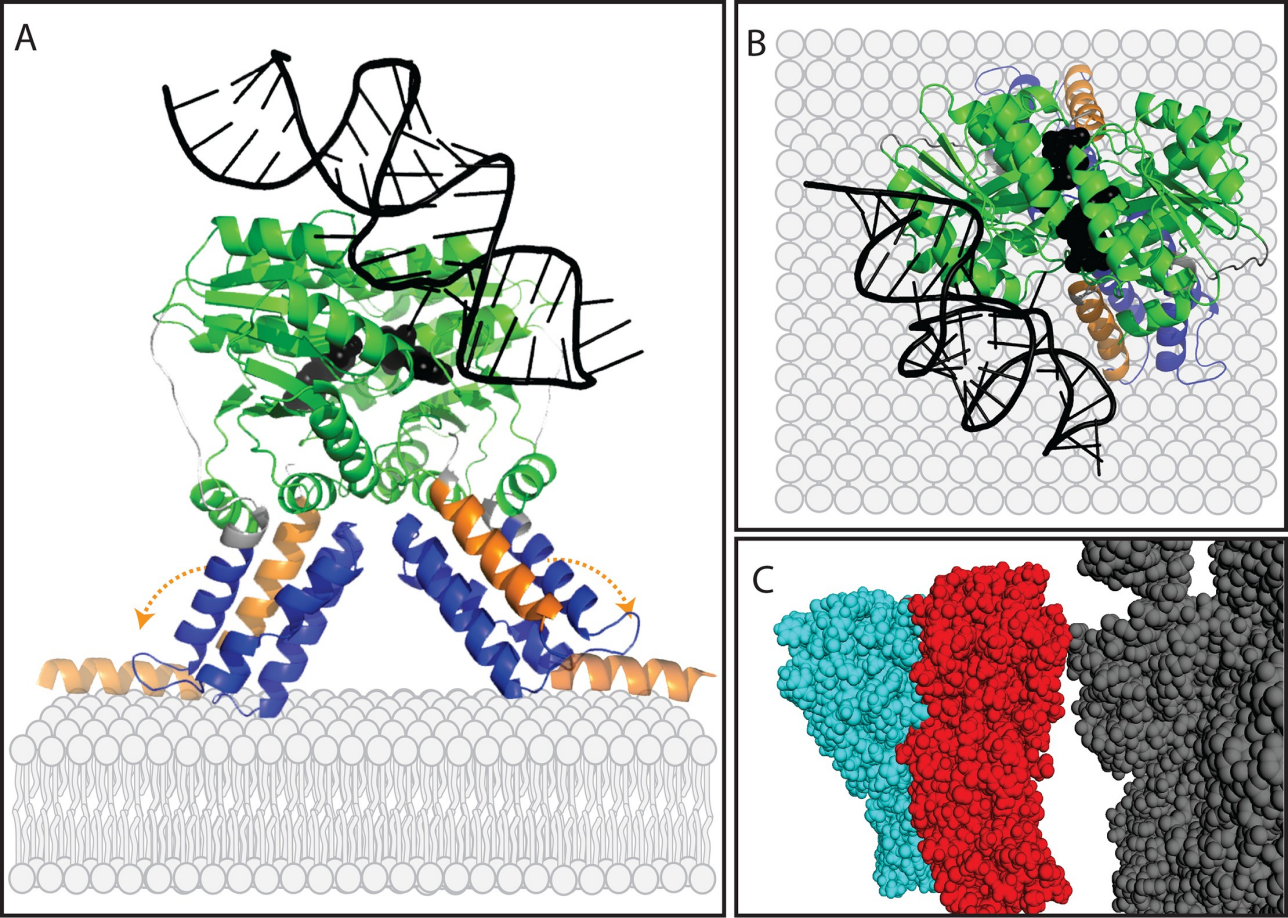
Predicted structural model of ancestral Ffh/FtsY. Predicted three dimensional structures of ancestral Ffh/FtsY are aligned to extant Ffh and FtsY proteins from x-ray diffraction structures (PDB IDs 4c7o and 5nco; [16,70]). In panels A and B, a potential homodimer of ancestral Ffh/FtsY is shown in complex with an SRP-RNA. The GTPase domains (green) form a GTP binding site that accommodates two GDP molecules (black), while the helical bundle domains (blue) and MTS domains (orange) are oriented toward the membrane. Orange arrows in panel A indicate the ability of the N-terminal MTS domains to reorient and attach to the interior surface of the membrane. Panel C depicts a potential homodimer of ancestral Ffh/FtsY (red and cyan) interacting with the 23S ribosomal subunit (gray).

The predicted structure of ancestral Ffh/FtsY also showed close structural alignment to both the Ffh protein (RMSD = 2.3) and FtsY protein (RMSD = 1.5) from *E*. *coli* that were solved in complex with GDP and an SRP-RNA tetraloop (PDB ID 4c7o). This structural alignment produced a model of ancestral Ffh/FtsY as a homodimer in complex with SRP-RNA and two guanosine nucleotides (Fig 5). In this model, the MTS domains are present in the N-terminal helices of each subunit and are located at the region of the complex that would likely make contact with the membrane. The GTPase domains make contact to the SRP-RNA and form a common active site that accommodates both GDP molecules. Taken together, these sequence- and structure-based characterizations of ancestral Ffh/FtsY suggest that this protein was, at minimum, capable of forming a homodimer in complex with an SRP-RNA, binding and hydrolyzing GTP, and binding to the membrane through MTS domains.

The predicted structure of SecY in the LUCA depicts a helix-rich protein (S1 Fig) with the greatest structural similarity to SecY of *Geobacillus thermodenitrificans* (Manachini et al. 2000) Nazina et al. 2001 emend. Coorevits et al. 2012 strain NG80-2 (RMSD = 0.49; PDB ID 6itcy; [45]). The structure-based function prediction from I-TASSER suggested that ancestral SecY in the LUCA was capable of membrane transport (GO: 0015450, GO:0055085) and was an integral membrane protein (GO:0016021; Table 2).

### Amino acid compositions inferred for ancestral proteins in the SRP system

The evolution of the canonical genetic code represents a stage in evolutionary history that likely occurred well before the time of the LUCA [46–51]. In order to test the hypothesis that the ancestral Ffh/FtsY arose in life prior to completion of the canonical genetic code, we analyzed the frequencies of late evolving amino acids in the reconstructed protein. Though there is disagreement about the exact chronology of the addition of amino acids to the genetic code, consensus between forty different published studies suggested the following order from earliest to latest is Gly/Ala, Val/Asp, Pro, Ser, Glu/Leu, Thr, Arg, Asn, Lys, Gln, Ile, Cys, His, Phe, Met, Try, and Trp [52]. A slightly more recent chronology based on phylogeny [53] is largely in agreement, especially regarding the latest five amino acids added to the code, which are predicted to be Cys, Phe, Tyr, Met, and Trp. A previous study by Fournier and Alm [54] analyzed the enzymes tyrosine aminoacyl tRNA synthetase (TyrRS) and tryptophan aminoacyl tRNA synthetase (TrpRS), which, like Ffh and FtsY, are paralogs that arose in an ancestor predating the LUCA. The authors found that Trp was missing from most of their sequence reconstructions for a pre-LUCA ancestor but became more likely in later nodes of their protein family tree, such as in the LUCA and the last shared ancestors of the bacterial and archaeal domains. Fournier and Alm [54] also performed ancestral sequence reconstructions of simulated sequences based on the WAG substitution matrix to show that the lack of Trp in the ancestor of the LUCA was not an artifact of Trp being a rare amino acid. The WAG substitution matrix is an empirically determined set of amino acid substitution rates from which sequences can be simulated to provide a base rate (i.e., typical rate) of amino acid usage for comparison against amino acid usage rates of reconstructed sequences.

**Fig 6:**
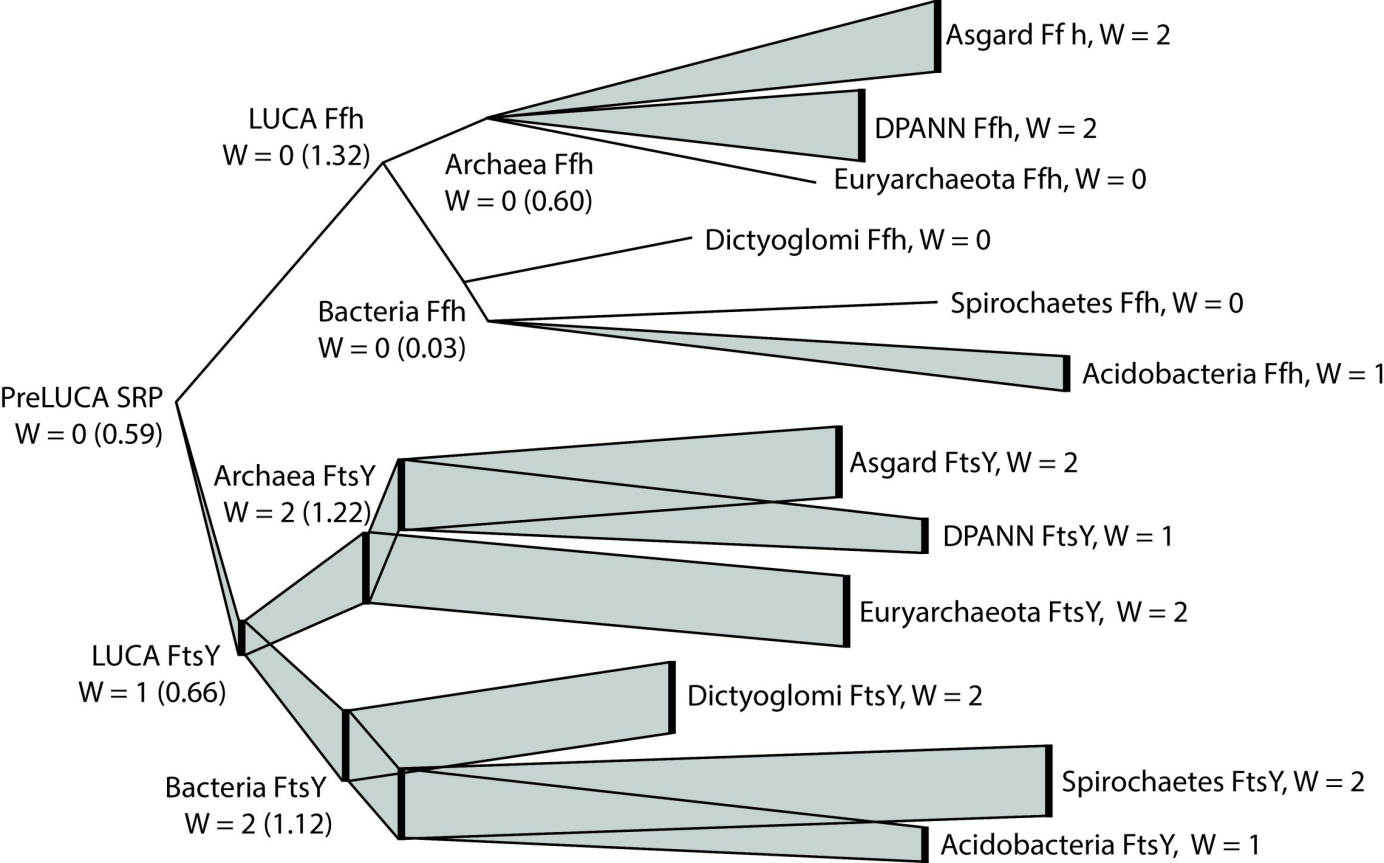
Inferred usage of tryptophan in the SRP system protein family from ancestral Ffh/FtsY to present. The tree topology and branch lengths are consistent with the SRP system tree shown in Fig 1. Branch widths correspond to the tryptophan usage at each node. For ancestral nodes, Trp usage is represented as the total Trp count in the consensus ancestral sequence and, in parentheses, the average Trp count across all sequence reconstructions at the corresponding node. Trp usage in extant Ffh and FtsY sequences is shown as median tryptophan usage in three phyla or superphyla of bacteria and three of archaea, which were chosen because their Ffh and FtsY sequences comprised clades without paraphyly or polyphyly (or nearly so) and represented the broadest sequence diversity within their respective taxonomic domains.

We performed a similar set of analyses to Fournier and Alm [54] in order to evaluate amino acid compositions during the evolutionary history of FtsY and Ffh. Our reconstructed sequence of ancestral Ffh/FtsY contained no Trp, while modern Ffh and FtsY proteins contain an average of 1.6 Trp, ranging from zero to five Trp in a single sequence. Reconstructed sequences of subsequent ancestors on the FtsY branch contained between one and two Trp, and ancestors on the Ffh branch contained no Trp (Fig 6). In contrast, ancestral sequence reconstructions based on the Ffh/FtsY tree (Fig 2) but containing terminal sequences simulated from the WAG substitution matrix (i.e., following Fournier and Alm [54]) yielded an average of 4.5 Trp at the node representing ancestral Ffh/FtsY. Therefore, the paucity of Trp in the ancestral Ffh/FtsY protein is not due to the rarity of Trp in modern proteins.

**Fig 7:**
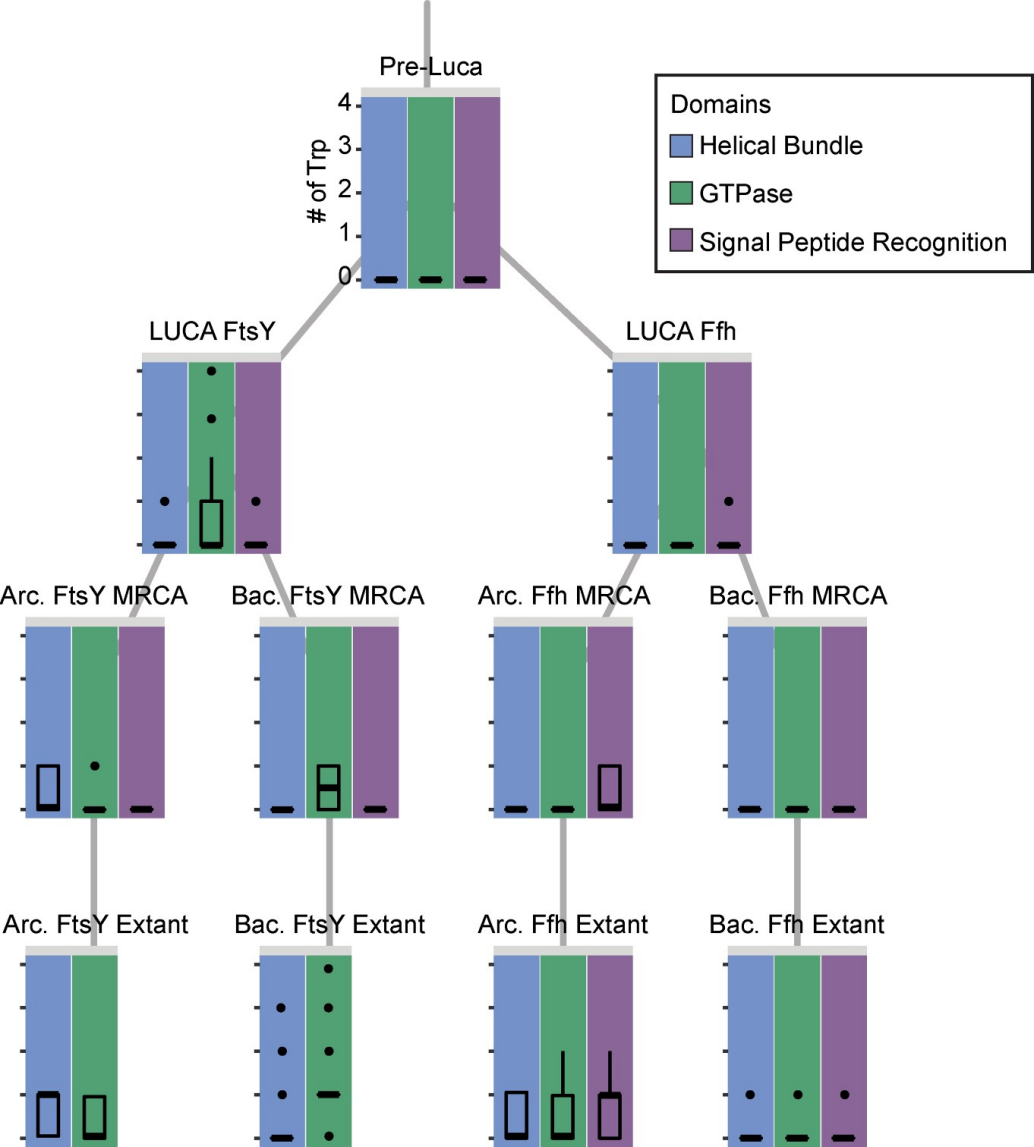
Incorporation of tryptophan into FtsY and Ffh protein domains through time based on ancestral sequence reconstructions and modern species. Box plots representing Pre-LUCA, LUCA, and bacterial (Bac) and archaeal (Arc) most recent common ancestors (MRCAs) are based on 100 ancestral sequence reconstructions using different subsets of the alignment of modern species. Subsets comprised one randomly selected sequence from each cluster identified in Clustal Omega (see Methods). For the modern species, tryptophan usage within extant proteins was determined only for Ffh and FtsY proteins, not homologs representing FlhA or the type III secretion system proteins. All box plots utilize the same scale for the y-axis presented adjacent to the set of plots for the pre-LUCA (top).

We also performed a finer resolution characterization of the evolutionary incorporation of Trp into the SRP system by examining the occurrences of this amino acid within protein domains through time at reconstructed nodes and in modern species. Interestingly, we found that Trp, if it was utilized at all in the SRP system of the LUCA, was most likely incorporated into the GTPase domain of FtsY, while, in Ffh, it could have been used within the signal peptide recognition domain, which is the only domain not shared between the two protein families (Fig 7). In the most recent common ancestor (MRCA) of Archaea, Trp may have been utilized within the signal peptide recognition domain of Ffh and in the helical bundle domain of FtsY. In contrast, the MRCA of Bacteria may not have used Trp at all within Ffh, but may have used it within the GTPase domain of FtsY. This is consistent with the modern usage among sampled species, in which Trp is more likely to be found in archaeal helical bundle domains and bacterial GTPase domains in FtsY. In Ffh, the archaeal lineage makes greater use of Trp in all three protein domains than Bacteria. It appears that the incorporation of Trp into the SRP system differed between the two protein families and between the two major prokaryotic evolutionary lineages.

**Fig 8:**
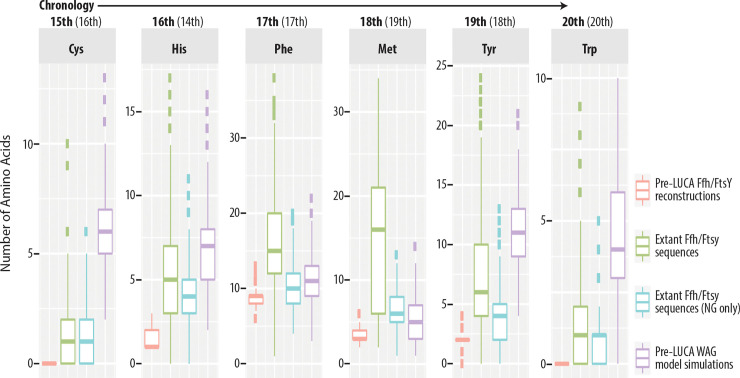
Paucity of late amino acids in ancestral Ffh/FtsY. The chronological order of the addition of late amino acids to the genetic code is taken from Trifonov [52] and Jordan *et al*. [53] (the latter shown within parentheses). Distributions of amino acid counts from the ancestral Ffh/FtsY sequence (red) represent 100 sequence reconstructions for the pre-LUCA node using the 10% gap threshold in FastML. Distributions of amino acid counts are shown for extant SRP system proteins (green) or only the region containing the helical bundle and GTPase domains that aligns to the ancestral Ffh/FtsY sequence (blue). When analyzing extant proteins, we excluded FlhA and type III secretion system proteins from the analysis. In all cases, the inferred ancestral Ffh/FtsY sequences contain fewer late amino acids than extant homologs. Reconstructions based on simulated sequences derived from the WAG model (purple) demonstrate that the paucity of late amino acids in ancestral Ffh/FtsY is not an artifact of the ancestral sequence reconstruction methods or the background rate of amino acid usage across proteins in general.

The above results regarding Trp should be interpreted with caution because of its rarity within proteins of the modern SRP system and, thus, due to the difficulty of inferring its prevalence in the past. Notably, many Ffh and FtsY proteins of modern organisms contain no Trp at all. We therefore extended this analysis to other late amino acids as per Trifonov [52] and Jordan et al. [53] (i.e., the two studies mentioned above on the chronological sequence of amino acid additions to the genetic code). In addition to Trp, the reconstructed sequence of ancestral Ffh/FtsY showed lower usage of all of the latest amino acids (Fig 8). Specifically, usages of the latest evolving amino acids in the ancestral Ffh/FtsY are much lower than in modern Ffh and FtsY proteins and much lower than predicted for the ancestor according to sequence simulations based on the WAG model. Taken together, these results suggest that the ancestral Ffh/FtsY dates back to the final stages of genetic code evolution, or at least, that it evolved at a time when the last amino acids to be incorporated into the genetic code were much less common within proteins.

### Early evolution of SRP/Sec membrane translocation

The cellular organization of organisms appears to have evolved sometime between the origin of life and the LUCA, a process that occurred roughly 3.5–4 billion years ago [55–60]. Within origins of life settings, the presence of amphipathic molecules with membrane-forming potential is well-documented [61,62]. However, in addition to the formation of membranes, cellularity also requires the ability to embed proteins in the membrane and secrete proteins through it. The evidence we present here constitutes the first thorough account of an ancient system of protein translocation and its evolution prior to the time of the LUCA. Specifically, we show that three central protein components of membrane translocation, FtsY, Ffh, and SecY, were all in place by the time of the LUCA and that they had functional and structural characteristics that were similar to their modern counterparts.

At this time, the available data in public databases do not indicate support for the presence of signal peptidase proteins or the SecA protein in the LUCA based on lack of orthologs well-represented among taxonomic domains and phyla or superphyla. These proteins perform seemingly essential functions; namely removing N-terminal signal sequences from secreted proteins and powering protein translocation through ATP hydrolysis. It may be the case that these proteins only enhance the SRP/Sec system and were not necessary to the system as it existed in the LUCA. Though we found that the Ffh sequence of the LUCA likely contained a signal recognition domain, this feature of the system may have been used only for internal signal sequences that need not be removed by a signal peptidase enzyme. However, more likely, these proteins may have undergone non-orthologous displacement [63] (or replacement) within the ancestral lineage of Archaea such that their presence in the LUCA can no longer be detected using phylogenetic methods. The process of non-orthologous gene displacement has been previously documented (e.g., [64,65]) and may explain other key differences in how the modern proteomes of Archaea and Bacteria accomplish fundamental processes such as DNA synthesis [66,67].

Beyond this account of the SRP/Sec system as it existed in the LUCA, we additionally show that the ancestral Ffh/FtsY protein that predated the LUCA had several functional characteristics found in modern Ffh and FtsY proteins and that this protein likely predates the completion of the canonical genetic code. Even though the ancestral Ffh/FtsY protein likely dates to this early stage in evolutionary history, it appears to have been capable of at least most of the functions performed by both proteins today. It had a membrane targeting sequence that could have bound to the membrane surface through electrostatic interactions in a manner similar to FtsY. It also had an N-terminal helical bundle and a GTPase domain, which, upon dimerization, created an active site that could bind and hydrolyze GTP (Fig 5). The GTPase domain, itself, belongs to the P-loop containing nucleoside triphosphate hydrolase superfamily, which is responsible for the majority of ATP and GTP hydrolysis within proteomes and is considered one of the most ancient tertiary structures and functional domains within all proteins [14,51,68]. It is unlikely that ancestral Ffh/FtsY contained a signal peptide binding domain, such as in Ffh, given that this domain is only present in sequences reconstructed with highly permissive inclusion of gaps.

Even without the ability to bind the signal sequence of a nascent protein, it is possible that ancestral Ffh/FtsY was capable of binding a ribosome during active translation, given that recent structural studies of the SRP-ribosome complex show contact between the large ribosomal subunit and the GTPase domain of Ffh [69,70]. Furthermore, though the main binding site for SRP-RNA is located in the M domain of Ffh [21], structural characterization of SRP-RNA co-crystalized with the Ffh and FtsY GTPase domains [16] suggests that ancestral Ffh/FtsY could have formed a homodimer in complex with an SRP-RNA (Fig 5). The presence of an MTS domain in the ancestral SRP system protein further suggests that this complex could have bound to a membrane surface.

Given that the ancestral Ffh/FtsY was able to bind the membrane but not able to bind signal peptides in a nascent protein, our results suggest that this ancestral protein was more like FtsY than Ffh and that a duplication event prior to the LUCA ultimately yielded the Ffh component of the SRP system. Under this scenario, Ffh appears to represent a neofunctionalization that likely increased efficiency of the SRP system through targeted signal sequence binding and, therefore, facilitating specialization of FtsY as a membrane bound receptor for Ffh, despite that FtsY appears to also retain ancestral capabilities to homodimerize [16]. Neofunctionalizations are sometimes regarded as resulting most frequently from an unobserved subfunctionlization step within the pathway of protein evolution following a duplication event [71,72], which, in this case, could be the loss of the membrane binding function prior to the gain of the signal peptide binding function.

Of course, it is possible that ancestral Ffh/FtsY was capable of all of the functions that are currently performed by both Ffh and FtsY (e.g., as in the reconstructions with 90% indel threshold; Fig 4, S8 File) and that SRP RNA and the Sec translocation channel were present alongside ancestral Ffh/FtsY in pre-LUCA organisms. However, even though we found that SecY was present in the LUCA, it is not a universal paralog, and, therefore, we cannot use phylogenetic analysis to infer its presence in organisms older than the LUCA. Similarly, the SRP RNA was likely present in the LUCA [73] and may potentially be much older than the LUCA [74], but it is not directly traceable to organisms predating the LUCA using phylogenetic methods.

**Fig 9:**
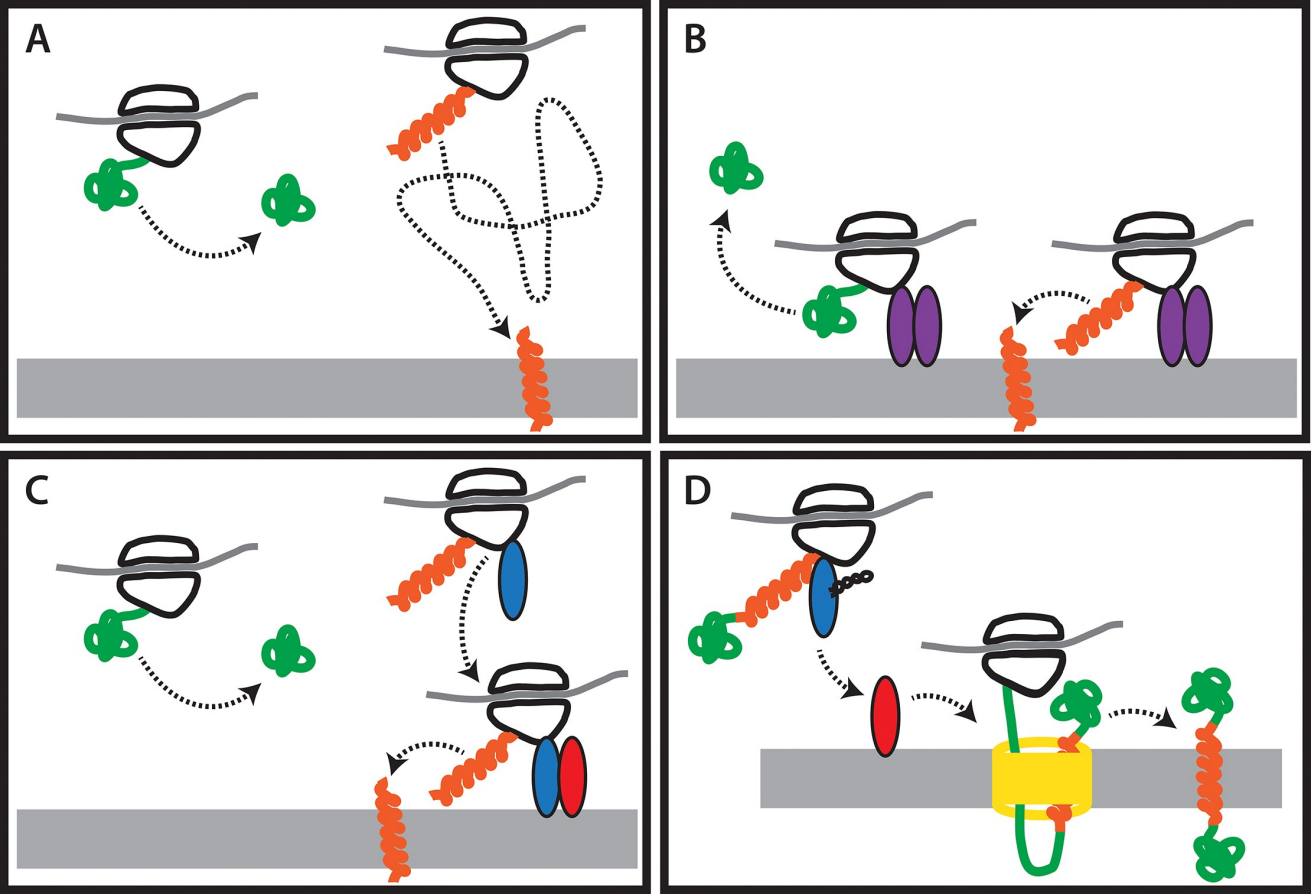
An incremental model for the evolution of the SRP membrane translocation system. A) At first, no components of the system exist. Ribosomes are present in the cytoplasm and some peptides are capable of spontaneous membrane translocation due to their chemical properties (orange), but only after they make their way to the membrane. B) The ancestral Ffh/FtsY protein (purple) evolves and acts to anchor ribosomes to the membrane, facilitating the spontaneous translocation of membrane proteins. C) The subsequent evolution of Ffh (blue) and the specialization of FtsY (red) as a receptor for Ffh allows one component of the system to bind ribosomes in the cytoplasm while they are synthesizing membrane proteins or secreted proteins. These ribosomes are then bound to the membrane surface through the interaction of Ffh and FtsY. D) The addition of the SecY membrane channel (yellow) to the system permits the translocation of large proteins that could not have done so spontaneously. Another equally likely order of events is that the SecY membrane channel preceded the divergence of the ancestral Ffh/FtsY protein into Ffh and FtsY.

Nevertheless, even without SecY and SRP RNA, ancestral Ffh/FtsY could have provided an early mechanism for delivering proteins to a membrane (Fig 9). The ancestral SRP system protein could have bound ribosomes through their GTPase domains. Ribosomes would then be temporarily bound to the interior of the membrane while they were translating. If the nascent peptide exiting the ribosome had certain chemical properties, it could spontaneously translocate across the membrane as is observed in some viral coat proteins (e.g.,[75,76]) and which is an important delivery system of peptide-based drugs [77]. If the nascent protein did not contain sequences capable of spontaneous membrane translocation, it would be released into the cytoplasm of the cell. The GTPase domain of the ancestral homodimer would regulate the formation and dissociation of the complex as it does in the Ffh/FtsY heterodimer, today. This system may not have required SRP RNA, which appears to function in response to the binding of a signal sequence.

This model (Fig 9) supported by our results suggests that the ancestral Ffh/FtsY, which predated the LUCA, could have served a similar function to its modern counterparts despite lacking some components of the modern system. The later functional refinement of the Ffh and FtsY proteins, along with the addition of the SRP RNA and SecY translocation would have significantly enhanced the membrane translocation process such that a highly sophisticated system had evolved by the time of the LUCA. As our evidence suggests, the earliest component of this system had evolved even before the final stages of genetic code evolution (Fig 7). This indicates that even very early life forms actively maintained cellular organization. Future work examining the early evolution of membrane proteins and of the enzymes that synthesized membrane components will further enhance our understanding of the most ancient forms of cellular organization.

## Materials and methods

### Sequence collection of FtsY, Ffh, and SecY homologs

We conducted BLASTp searches to obtain homologous proteins in four archaeal and 18 bacterial superphyla or phyla that were represented by at least one complete genome in the NCBI database and well-characterized taxonomically (i.e., excluding *incertae sedis* despite the relevance of poorly characterized bacterial lineages to the tree of life [32]). We performed the BLASTp using query sequences of *Escherichia coli* (Migula 1895) Castellani and Chalmers 1919 (Proteobacteria; accessions P10121, P0AGD7, P0AGA2 of the SwissProt database [78], a model bacterium, and *Haloferax volcanii* (Mullakhanbhai and Larsen, 1975) Torreblanca et al., 1986 (Euryarchaeota; accessions Q977V3, D4GYW6, Q977V2), an emerging model archaean [79,80]. Prior to performing the BLAST searches, we verified that the representative sequences of FtsY, Ffh, and SecY in *E*. *coli* and *H*. *volcanii* were reciprocal top hits of each other within their respective genomes from the RefSeq database ([81]; accessions: ASM584v2, ASM2568v1) based on e-values. We conducted the BLASTp searches using systematic taxonomic sampling within superphyla or phyla according to the NCBI taxonomic database [82,83] to account for diversity across the tree of life. Preliminary BLAST search results and surveys of the literature suggested that the flagellar synthesis protein, FlhA, and the EscV/YscV/HrcV genes of the type III secretion system, evolved within the SRP system protein family [84]. Therefore, we also performed searches for FlhA or type III secretion system proteins using accessions P76298, A0A2J6N6Z4, O85633, and A0A2K3J983 as query sequences. After obtaining a dataset containing 20 (or as many as available) taxonomically diverse proteins of each type from each phylum or superphylum, we performed reciprocal BLAST searches to ensure their orthology with the target proteins.

For all BLAST searches, we accepted only those results with e-values of 0.0001 or lower and query and target coverage of at least 75%. Where possible, we selected different genera or, less ideally, different species from among the top hits for a total of 20 sequences or the maximum number available meeting our criteria. We performed reciprocal BLAST searches of the obtained proteins into the genomes of *E*. *coli* and *H*. *volcanii* from RefSeq ([81]; accessions: ASM58ASM584v2, ASM2568v1) to ensure their orthology with the target proteins and discarded sequences for which the top hit was not at the expected locus.

### Phylogenetic analysis

For the final protein datasets of the SRP system and SecY, we aligned sequences in Geneious v. 11.1.5 [85] using MAFFT [86–88] with a gap open penalty of 3.0 and extension penalty of 1.0. We allowed Geneious to automatically select the best algorithm within MAFFT for our data. We adjusted the alignments using MAFFT on either side of highly conserved blocks as well as performing manual adjustments.

We conducted Bayesian phylogenetic inference using ExaBayes [89] on the Comet supercomputing cluster of XSEDE for the dataset of sequences representing SRP system, including FlhA and type III secretion system proteins, and separately for the dataset of sequences representing SecY. The analyses for each dataset comprised two independent runs of 20 million generations with sampling every 5000 generations. We applied the WAG amino acid substitution matrix [90] based on preliminary analyses under mixed models in ExaBayes and MrBayes 3.2 [91–93]. Following the analyses, we assessed convergence of independent runs and stationarity according to ESS values > 200 in Tracer v1.6 [94]. We performed 10% burnins for each run and combined the remaining trees in Log Combiner of the BEAST v2.0 package [95,96]. From among the combined trees, we obtained the maximum clade credibility tree using Tree Annotator, also part of the BEAST package, with median branch lengths and no posterior probability cut-off.

The MAFFT sequence alignment for the SRP system comprised 1666 amino acids, and showed large gaps, especially in the N- and C- terminal regions. Within the N-terminal regions, the gaps largely occurred because some FtsY proteins have extended, non-conserved sequence in this region, while in the C-terminal regions, Ffh proteins contain the signal peptide binding motif that is lacking in FtsY. The SecY alignment, having a length of 835 amino acids, also contained large gaps, such as may be anticipated when aligning sequences across domains of life. To ensure that retaining gap-rich regions did not significantly impact tree topologies, we also performed phylogenetic analyses on matrices, in which we removed highly gapped regions. To remove these regions, we applied Gblocks [97] to both the full SRP and SecY alignments.

Within Gblocks, we set the minimum number of sequences for a conserved or flanking position to the minimum allowable (ca. just over half the number of total sequences in the alignment; 470 of 938 sequences for SRP and 178 of 355 for SecY). We set Gblocks to accept up to 100 contiguous non-conserved positions, a minimum length block of two, and to allow any number of positions within the conserved block to contain gaps. This represents a relaxed set of parameters [98] for selecting conserved blocks of protein sequences, and more conservative settings yielded no blocks in preliminary analyses. The resulting alignment of the SRP system proteins (S9 File) comprised 706 sites consisting of 49.0% gaps (compared to 68.3% in the complete alignment). Through comparison with the complete alignment for the SRP system, we confirmed that the use of Gblocks resulted primarily in the trimming of the N- and C-terminal regions that are present only in some FtsY and most Ffh, respectively. For SecY, the alignment based on Gblocks (S10 File) contained 667 sites and 38.6% gaps (compared to 43.0% in the complete alignment).

We performed phylogenetic analyses for the trimmed alignments using ExaBayes and summarized the resulting trees as described above for the complete alignments. We visually compared the trees resulting from the Gblocks and complete alignments using the cophylo function of the Phytools library [99] for R (S2 and S3 Figs). We also investigated the posterior probabilities for nodes between the Gblocks and complete alignment trees using cumulative fraction plots (S4 and S5 Figs) and by performing one-tailed t-tests (assuming equal variance based on prior F-tests). These results show that, in the SecY tree, only a few terminal taxa occur at different positions in the trimmed, Gblocks alignment compared to the complete one (S3 Fig). For SRP, several superphyla or phyla are in different positions (S2 Fig). However, in both cases, the use of Gblocks did not change major findings, such as low rates of inter-domain HGT, overall tree shape, or, within the SRP system, the relationships among proteins. Moreover, the use of Gblocks significantly (*α* = 0.05) lowered support overall across the tree topologies (S4 and S5 Fig), as speculated in prior studies (e.g., [100]). Thus, we utilized the complete alignments for downstream analyses.

### Ancestral sequence reconstruction

We performed ancestral state reconstructions using FastML [101]. Preliminary analyses of sequence reconstructions in FastML revealed that ≥300 input sequences yielded an error, which appeared related to very small values rounded to zero (i.e., a precision error). Therefore, we reduced the size of our datasets for ancestral sequence reconstruction using Clustal Omega [102] with soft bounds on the number of sequences per cluster; five for the SRP system and three for SecY. We expected these soft bounds to yield between 200–300 clusters for each dataset, and we obtained 286 clusters for the SRP system (S1 File) and 166 for SecY (S2 File). To avoid biases or anomalies in sequence selection within the clusters, we assembled 100 datasets for the SRP system and SecY by randomly selecting an accession from each cluster. We performed sequence reconstruction in FastML on each of the 100 datasets and assembled the results for each ancestral node of interest in the phylogeny into a consensus sequence for downstream analyses.

In FastML, we conducted marginal sequence reconstructions over the SRP and SecY phylogenies with maximum likelihood inference of gaps, and we used our maximum clade credibility trees of the SRP system and SecY as guides rather than allowing FastML to reconstruct internal guide trees. For each analysis, we pruned the input tree to represent the 100 selected sequences. For the ancestral sequence reconstructions, our nodes of interest were the LUCA node in SecY that is also the root of the tree and, in the SRP system tree, the LUCA nodes of Ffh and FtsY, and the ancestral Ffh/FtsY representing the root of the tree. For each of these nodes, we obtained a consensus sequence from the 100 results using the simple majority method in the seqinr library for R [103].

### Protein function prediction of ancestral sequences

In addition to identifying conserved domains and motifs of the reconstructed ancestral sequences in the PFAM database, we also identified potential MTS domains, which are not represented in PFAM, in ancestral Ffh/FtsY by alignment to the *E*. *coli* sequence in which this domain was first characterized [Protein Data Bank (PDB; [104,105]) ID: 2yhs; [22]] using the ClustalW2 webserver (https://embnet.vital-it.ch/software/ClustalW.html; [106]) on default settings. We calculated the isoelectric point of the MTS domain of *E*. *coli* and predicted MTS domains in our reconstructed ancestral sequence using the ProtParam webserver [107].

To complement the sequence-based approaches, we also predicted the three-dimensional protein structures of the ancestral proteins in I-TASSER [40]. In addition to predicting three-dimensional structures, I-TASSER also finds the most structurally similar proteins in the PDB according to the root mean square deviation (RMSD) of the distance between atoms in structural alignments and maps proteins to their most probable gene ontology (GO) terms [108,109] using the structure-based function prediction database, BioLiP [110]. To better assess interactions of ancestral Ffh/FtsY with GTP and SRP RNA, we aligned the top-ranked structural model from I-TASSER to an x-ray diffraction structure of the conserved NG domains of both Ffh and FtsY, bound as a heterodimer and co-crystallized with GDP and the SRP-RNA tetraloop (PDB ID: 4c7o; [16]) using the "align" function in MacPyMOL (Schrödinger LLC.; [111]).

### Amino acid composition analysis of ancestral sequences

We compared the frequencies of the most recently added amino acids, Cys, His, Phe, Met, Tyr, and Trp in the reconstruction of ancestral Ffh/FtsY to the frequencies of their modern descendants represented by our dataset of protein sequences for the SRP system. These latest additions to the canonical genetic code were based on consensus between the influential chronology published by Trifonov [52], which itself is based on consensus of 40 prior studies, and a more recent chronology published by Jordan et al. [53]. We performed two comparisons; one of the complete alignment, including FlhA and the type III secretion system proteins, and another in which we removed FlhA and type III secretion system protein sequences and trimmed the remaining alignment to contain only the conserved NG domains of FtsY and Ffh. Additionally, for Trp, which is widely believed to be the latest amino acid added to the genetic code [52,53], we also analyzed its frequency in reconstructed sequences of the archaeal and bacterial crown clades within the FtsY and Ffh clades of the SRP system (i.e., based on the full alignment; see above).

We sought to ensure that the frequency of late arising amino acids in ancestral Ffh/FtsY was not an artifact of the method of ancestral sequence reconstruction in FastML. Therefore, we simulated sequences across the phylogenetic tree of the SRP system in the R package phangorn [112] under the WAG model. We used the WAG model as a standard against which to compare because it was the model selected to represent sequence evolution of the SRP system in the RMC analysis and because we based our analyses of ancestral amino acid composition on Fournier and Alm [54], who also used the WAG model. We performed 100 simulations, in which we generated new datasets with the same total number of sequences (938; including FlhA and the secretion system) of the same length as the existing alignment (1666 amino acids). Subsequently, we placed gaps in the simulated sequences for each terminal taxon at the loci where they occurred in the existing alignment, thereby using the empirical data to determine the evolution of indels rather than modeling them. We used the newly simulated sequences with indels as alignments to infer ancestral sequences in Fast ML. We performed these analyses as above by generating 100 smaller datasets using Clustal Omega, generating only one alignment of reduced size for each dataset of simulated sequences. For each reduced alignment, we pruned the SRP system tree accordingly so that it could be applied as a guide. In FastML, we estimated gaps at a 10% threshold, and we regarded the consensus sequences at internal nodes and the root node (i.e., representing an ancestor of the LUCA) to be the simulated ancestral sequence reconstructions.

## Supporting information

S1 File Output of Clustal Omega showing 938 proteins representing the SRP system organized into 286 groups. (TXT)

S2 File Output of Clustal Omega showing 355 proteins representing SecY organized into 166 groups. (TXT)

S3 File Complete gapped alignment of 938 sequences representing the SRP system used in this study. Sequence names follow the format gene_domain_phylum_Accession#, where gene refers to either FtsY, Ffh, FlhA or the secretion system (SS), domains and phyla or superphyla are according to NCBI taxonomy, and accession numbers are from the NCBI nr protein database.
(TXT)

S4 File Complete gapped alignment of 938 sequences representing the SRP system used in this study. Sequence names follow the format gene_domain_phylum_Accession#, where gene is always SecY, domains and phyla or superphyla are according to NCBI taxonomy, and accession numbers are from the NCBI nr protein database.
(TXT)

S5 File Maximum clade credibility tree resulting from Bayesian analysis of SRP system proteins (see S3 File) in ExaBayes showing branch lengths and posterior probability support values. (TXT)

S6 File Maximum clade credibility tree resulting from Bayesian analysis of SecY proteins (see S4 File) in ExaBayes showing branch lengths and posterior probability support values. (TXT)

S7 File Ancestral sequences reconstructions for SecY and the SRP system. For the SRP system, we show results for three different cutoffs for inferring gaps: 10%, 50%, and 90%. For SecY, we used only the cut-off of 50%. In each case, there are 100 sequences resulting from analyses of reduced datasets derived from the clusters predicted in Clustal Omega (see S1 and S2 Files).
(TXT)

S8 File Consensus sequences of 100 ancestral reconstructions of reduced datasets of SecY and the SRP system. For the SRP system, we show consensus sequences for 10%, 50%, and 90% thresholds for inferring gaps, while for SecY, we show only the 50% threshold.
(TXT)

S9 File Alignment of 937 sequences representing the SRP system used in this study processed in Gblocks. Sequence names follow the format gene_domain_phylum_Accession#, where gene refers to either FtsY, Ffh, FlhA or the secretion system (SS), domains and phyla or superphyla are according to NCBI taxonomy, and accession numbers are from the NCBI nr protein database. Note that the sequence SS_Archaea_TACK_TBR20608 is present in the ungapped alignment (S3 File and Fig 2) but not the alignment processed in Gblocks, which led to the removal of all sites containing this sequence.
(TXT)

S10 File Alignment of 938 sequences representing the SRP system used in this study processed in Gblocks. Sequence names follow the format gene_domain_phylum_Accession#, where gene is always SecY, domains and phyla or superphyla are according to NCBI taxonomy, and accession numbers are from the NCBI nr protein database.
(TXT)

S1 Table Comparison representative sequences of FtsY (light colors) and Ffh (dark colors) among *E*. *coli* (blue) and *H*. *volcanii* (yellow) annotated for GO terms representing Biological Function (Green), Cellular Process (Purple), and Cellular Component (Orange). Annotations are according to Uniprot for accessions P10121, P0AGD7, Q977V2, and D4GYW6. Adjacent sequences match between organisms and bold highlighting indicates that the ancestral Ffh/FtsY was also annotated with the term.
(XLSX)

S1 Fig Predicted structure of SecY in the LUCA according to I-TASSER based on ancestral sequence reconstruction. The image represents the most highly supported of two models based on a C-score of 1.57.
(PNG)

S2 Fig Maximum clade credibility trees of the SRP system resulting from analysis of the complete, gapped alignment (right) and Gblocks-processed alignment (right) in ExaBayes. Trees are rotated to minimize crossings of lines that connect identical accessions between trees. This provides a visual representation of the similarity between the trees resulting from the two analyses. The visual was generated using Phytools in R.
(PDF)

S3 Fig Maximum clade credibility trees of SecY resulting from analysis of the complete, gapped alignment (right) and Gblocks-processed alignment (right) in ExaBayes. Trees are rotated to minimize crossings of lines that connect identical accessions between trees. This provides a visual representation of the similarity between the trees resulting from the two analyses. The visual was generated using Phytools in R.
(PDF)

S4 Fig Cumulative distribution plot and t-tests comparing posterior probabilities of nodes based on ExaBayes analyses of complete, gapped alignments and alignments with Gblocks processing of SRP system proteins. (PDF)

S5 Fig Cumulative distribution plot and t-tests comparing posterior probabilities of nodes based on ExaBayes analyses of complete, gapped alignments and alignments with Gblocks processing of SecY. (PDF)
