# Supplementary figures and images for: The very early evolution of protein translocation across membranes
Source: PLoS Comput Biol. 2021 Mar 8;17(3):e1008623. doi: 10.1371/journal.pcbi.1008623 (PMC7987157; doi:10.1371/journal.pcbi.1008623)

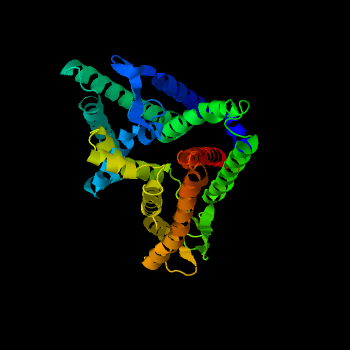

Supplement: S1 Fig — The image represents the most highly supported of two models based on a C-score of 1.57. (PNG) [file pcbi.1008623.s012.png]

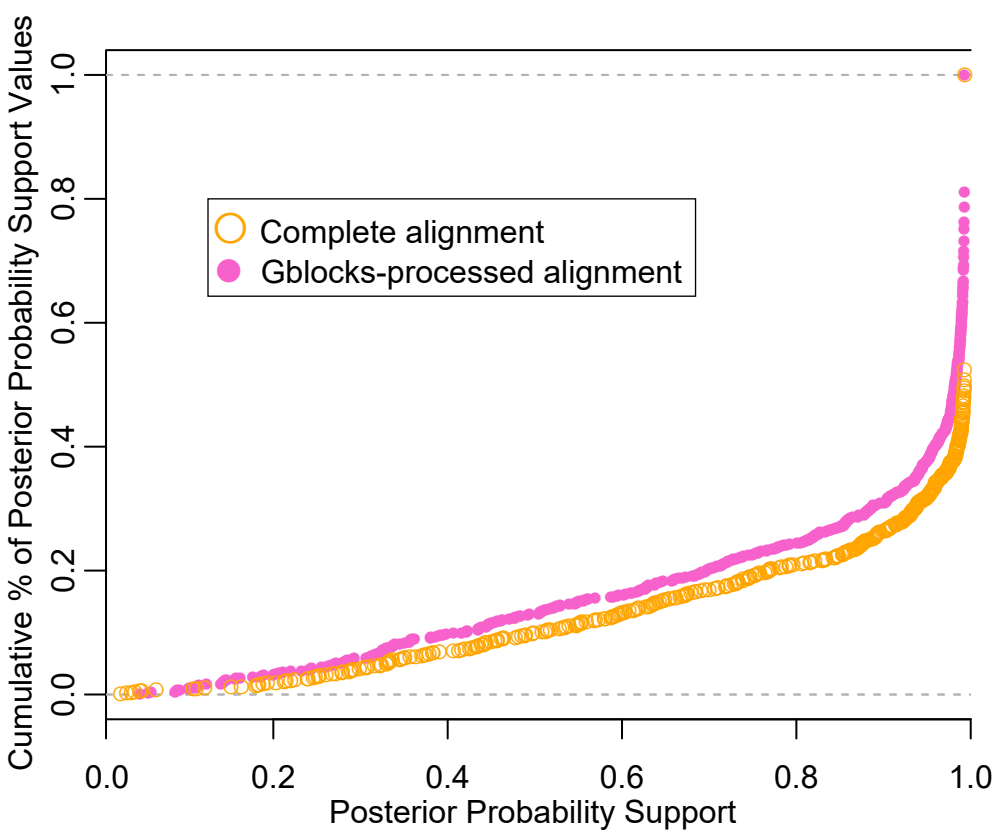

Comparison of posterior probabilities using unpaired t-test

H: ○ > ●

$T_{\text{stat}} = 2.69$

$p = 0.004$

Supplement: S4 Fig — (PDF) [file pcbi.1008623.s015.pdf]

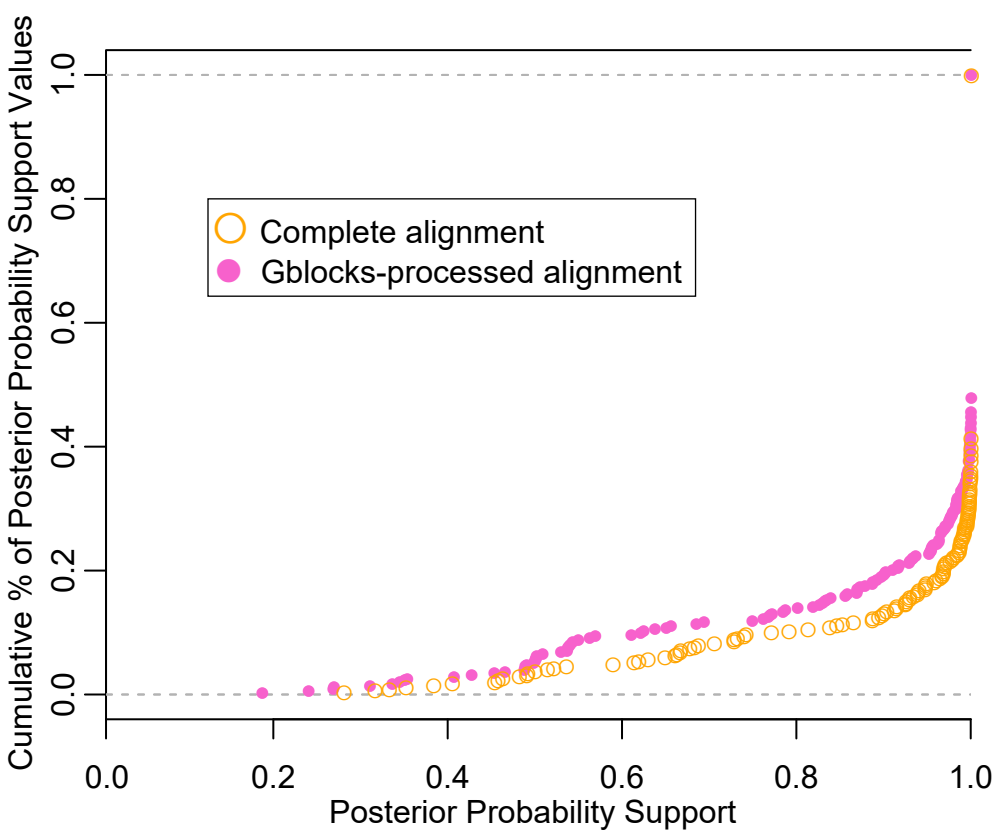

Comparison of posterior probabilities using unpaired t-test

H: ○ > ●

$T_{\text{stat}} = 2.18$

$p = 0.015$

Supplement: S5 Fig — (PDF) [file pcbi.1008623.s016.pdf]
