# Supplementary material for: The very early evolution of protein translocation across membranes
Source: PLoS Comput Biol. 2021 Mar 8;17(3):e1008623. doi: 10.1371/journal.pcbi.1008623 (PMC7987157; doi:10.1371/journal.pcbi.1008623)

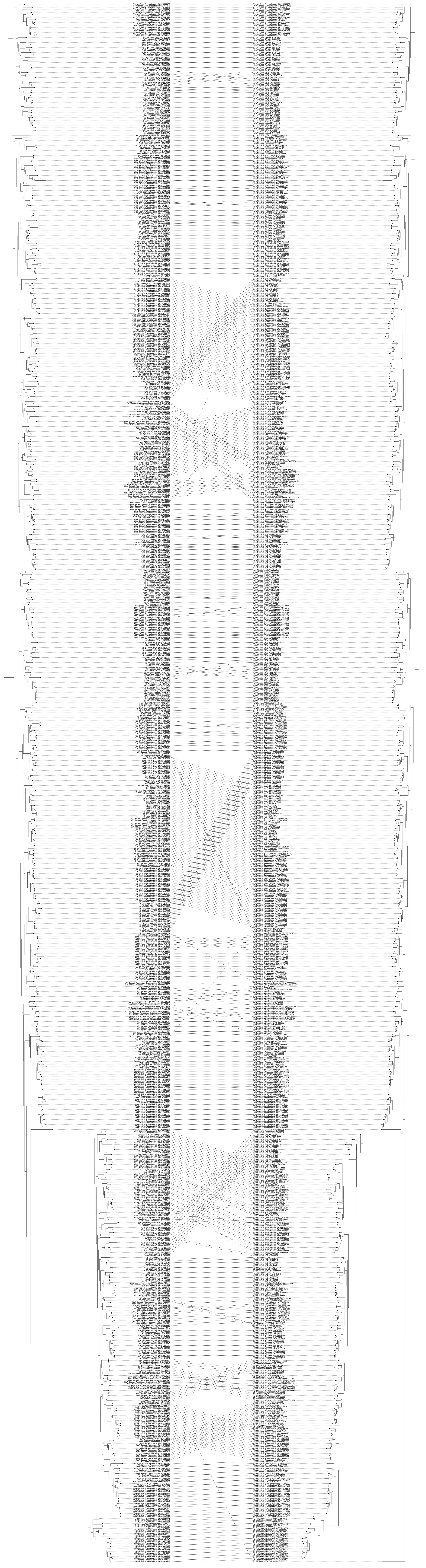

Supplement: S2 Fig — Maximum clade credibility trees of the SRP system resulting from analysis of the complete, gapped alignment (right) and Gblocks-processed alignment (right) in ExaBayes. Trees are rotated to minimize crossings of lines that connect identical accessions between trees. This provides a visual representation of the similarity between the trees resulting from the two analyses. The visual was generated using Phytools in R. (PDF) [file pcbi.1008623.s013.pdf]
